# Enacting quality improvement in ten European hospitals: a dualities approach

**DOI:** 10.1186/s12913-020-05488-9

**Published:** 2020-07-16

**Authors:** Francisco G. Nunes, Glenn Robert, Anne Marie Weggelaar-Jansen, Siri Wiig, Karina Aase, Anette Karltun, Naomi J. Fulop

**Affiliations:** 1 ISCTE-IUL, Lisbon University Institute, BRU-IUL, Avenida das Forças Armadas, 1649-026 Lisbon, Portugal; 2 King’s College London, Strand, London, WC2R 2LS UK; 3 iBMG – Erasmus University Rotterdam, Postbus 1738, 3000 DR Rotterdam, The Netherlands; 4 SHARE-Centre for Resilience in Healthcare, Faculty of Health Sciences, University of Stavanger, N-4036 Stavanger, Norway; 5 The Jönköping Academy for Improvement of Health and Welfare, School of Health and Welfare and Department of Supply Chain and Operations Management, School of Engineering, Jönköping University, PO Box 1026, SE-551 11 Jönköping, Sweden; 6 Department of Applied Health Research, University College London, 1-19 Torrington Place, London, WC1E 7HB UK

**Keywords:** Quality improvement, Organizational change, Paradoxes, Dualities

## Abstract

**Background:**

Hospitals undertake numerous initiatives searching to improve the quality of care they provide, but these efforts are often disappointing. Current models guiding improvement tend to undervalue the tensional nature of hospitals. Applying a dualities approach that is sensitive to tensions inherent to hospitals’ quest for improved quality, this article aims to identify which organizational dualities managers should particularly pay attention to.

**Methods:**

A set of cross-national, multi-level case studies was conducted involving 383 semi-structured interviews and 803 h of non-participant observation of key meetings and shadowing of staff in ten purposively sampled hospitals in five European countries (England, the Netherlands, Portugal, Sweden, and Norway).

**Results:**

Six dualities that describe the quest for improved quality, each embracing a seemingly contradictory feature were identified: plural consensus, distributed connectedness, orchestrated emergence, formalized fluidity, patient coreness, and cautious generativeness.

**Conclusions:**

We advocate for a move from the usual sequential and project-based and systemic thinking about quality improvement to the development of meta-capabilities to balance the simultaneous operation of opposing ideas or concepts. Doing so will help hospital managers to deal with major challenges of change inherent to quality improvement initiatives.

## Background

### Quality improvement efforts with unclear results

As key players in the health care field, hospitals become involved in numerous quality improvement (QI) initiatives [1, 2]. Despite a general impetus to improve care, the results of these efforts are not clear. If some initiatives have shown positive results on patient outcomes [3], others have failed to show significant improvement [4], generated specific negative results [5], or appear to be generalized disappointments, like the case of lean based QI interventions [6]. Nevertheless, improvement is a necessity. According to the World Health Organization [7] up to 40% of all care spending is wasted through inefficiency and of 421 million hospitalizations globally each year 10% result in harm to patients. The room for improvement remains impressive.

As QI implies some sort of organizational change, in order to explain these mixed results and advance the effectiveness of QI interventions, scholars have turned their attention to the role of organizational features in the change process [8] and securing improvement [9]. Research exploring the impact of organizational factors on efforts to increase the effectiveness of improvement efforts abound, and a profusion of concepts, terminology, intervention strategies and indicators are now available. This prolixity led some authors to propose meta-models able to offer a more comprehensive overview of change processes and aimed at providing guidance in change management. The Consolidated Framework for Implementation Research (CFIR) [10] and The Model for Understanding Success in Quality (MUSIQ) [11] represent such integrative efforts.

The MUSIQ was built as a result of a systematic review of 47 empirical studies about the influence of context in QI initiatives [11]. Its revision highlighted the relevance of organizational elements, especially leadership from top management, culture, information systems and experience in QI, but also physician involvement, motivation to change, existence of resources for QI, and QI team leadership. Subsequently, the results of this systematic literature review were used as input for a panel of experts to establish relationships between constructs and thereby developed MUSIQ as an integrative model of previous research about QI success.

The CFIR was built from an implementation science perspective [10]. Researchers reviewed 19 models of effective change in health services and suggested a comprehensive framework covering five major domains: intervention characteristics, outer setting, inner setting, characteristics of individuals involved, and the process of implementation. These high-level domains contain several organizational elements, at least partially coincident with the MUSIQ framework, including culture, structural characteristics, and leadership engagement or commitment.

Together, CFIR and MUSIQ represent notable integrations of previous knowledge about change dynamics in health care organizations. Both models provide a more comprehensive picture of key organizational elements, and their relationships, that influence successful change as well as offering reflective tools to enable practitioners to conduct more fruitful improvement projects. However, in this process of knowledge integration, we suspect that some central features of health care organizations have lost their relevance which may weaken the sense-making and pragmatic value of these models as currently constituted.

More precisely, we believe that both models are not sensitive to the tensional nature of organizations in general, and health care providers in particular, which limits their value as a framework to understand and effectively guide improvement. Health service providers are challenged to deal with the contradictory pressures of delivering better care for more people at lower costs [12] and, at the organizational level, managers are dealing with these pressures using locally developed strategies [13]. On the other hand, QI initiatives require some sort of departure from the status quo and organizational change inevitably entails tensions, competing requirements, contradictions, and dilemmas [14]. Current approaches to QI tend to ignore these features. In a context of increasingly demanding resources, managing tensions arising from external pressures to enact multiple conflicting strategies, while addressing internal pressures to enact multiple cultures and identities, become core features of the change dynamics in contexts like health care [15]. In our view, the paradox approach to organizational sustainability offers appropriate insights about dealing with usual tensions surfacing in healthcare organizations who try to change under conditions of plurality and resource scarcity.

### A dualities approach of organizational sustainability: the paradox view

The idea that paradoxes are central constructs in understanding organizational dynamics has recently grasped the attention of numerous researchers [16, 17]. At the heart of this perspective lies the idea that organizations are best described, understood, and managed if we highlight, recognize, and embrace their persistent, pervasive, and interwoven tensions [18].

Tensions come from the co-existence of oppositional demands, or dualities, like flexibility and control, differentiation and integration, stability and change. These contradictory elements are best viewed not as choices to be made under certain circumstances, the “either/or” approach suggested by classic systemic and contingency perspectives, but requiring a shift to a “both/and” perspective. In one specific paradox, tension comes from the fact that seen in isolation, each element makes perfect sense, but when both elements appear at once, the co-existence seems illogical. Despite this irrationality, both elements are indivisibly interconnected and interdependent, and this dynamic of contradiction and interdependence continues over time establishing a cyclical relationship, in the sense that each element is required to constitute the other. More parsimoniously, a paradox is defined as “persistent contradiction between interdependent elements” ([17], p. 6).

According to the dualities approach, paradoxes can stay dormant within a specific organization or become active under certain circumstances. Tensions become more salient when organizations are plural, are required to change permanently and face resource scarcity [19]. Because hospitals are described as plural organizations forced to enact different cultures and identities [15], facing resource scarcity [20] and continuously changing to improve their level of care, they become contexts in which the salience of persistent tensions becomes the norm and not the exception, calling for the development of capabilities enabling organizations to deal with tensions. When managers engage in proper strategies to handle the paradoxical tensions inherent to organizational life, they are crafting capabilities that contribute to organizational sustainability, seen as a dynamic process by which organizations become able to respond to various internal and external stakeholders and define their success in the short and long term [21–23].

To state that improving the quality of the service provided by hospitals is better achieved if we adopt a dualities perspective says nothing about what tensions are in place when hospitals get involved in improvement efforts, or what leaders can do to take advantage of these tensions. In addition, the paradox perspectives provide limited answers to the fundamental concern of determining specific tensions in the context of health care services. This calls for inductive research that, drawing upon specific improvement efforts undertaken by hospitals, enables us to identify sources of tension coming from oppositional demands and corresponding reconciliation capacities, here named meta-capabilities. This paper therefore addresses the following research question:

*To which organizational dualities should hospital managers attend in order to improve the quality of health care?*

To answer this question we used data collected in case studies conducted in ten European hospitals and we inductively identified six dualities underlying the quest for improved quality, each embracing a seemingly contradictory feature: plural consensus, distributed connectedness, orchestrated emergence, formalized fluidity, patient coreness, and cautious generativeness. By proposing these six dualities we are contributing to the literature on how health care organizations strive to improve in a context constrained by lack of resources and great pressure to achieve better outcomes.

## Methods

### Study design

This study is based on the data collected within the Quality and Safety in European Hospitals (QUASER) project. QUASER studied quality as a multi-dimensional concept, involving patient safety, patient experience and clinical effectiveness, and used a multi-level case study design in 10 hospitals in five European countries: The Netherlands, Norway, Sweden, Portugal and England. The QUASER research protocol [24] contains a detailed description of the research approach. As a multilevel study, three levels of analysis were considered: macro, meso and micro. The macro-level comprises the usual contextual factors surrounding hospitals, in this case, each of the five national healthcare systems. The meso-level refers to the organizational level, the hospital, in this case. The micro-level corresponds to specialized units, like maternities or intensive care units, the ones where frontline employees are nested. Hospitals were selected in accordance with a protocol in order to study hospitals at different stages of the quality journey, a process thoroughly discussed elsewhere [25]. Table 1 provides a brief description of the study hospitals.

**Table 1:** Main characteristics of the hospitals studied

	Sweden		Portugal		Norway		England		The Netherlands	
	A	B	A	B	A	B	A	B	A	B
Teaching	yes	yes	yes	no	yes^a^	yes	yes	no	yes	yes
Beds	506	642	1300	585	300	1100	2200	1025	709	536
Staff	3300	4082	1772	1343	2336	11,000	12,000	7500	3677	2649

^a^This hospital has teaching status for nurses but not for any other professional groups

In each country A is the hospital which performed relatively well against a set of predefined quality indicators (B is the ‘less well performing’ hospital). Although different countries collect different indicators, use different definitions of the same indicators, use different data collection requirements, put in place different levels of aggregation of data, and have different hospital accreditation and licensing [25], at the national level hospitals were classified as A or B according to locally accepted indicators. The most common indicators were surgical site infection rates, specific mortality rates, caesarean section rates, and hip fractures treated in set time. In the ‘A’ hospitals, two clinical micro systems were analyzed in depth covering a range of services: maternity, oncology, orthopaedics, elderly care, intensive care, and geriatrics. The rationale for studying two micro systems in each hospital was to grasp the influence of processes located at different levels of analysis on quality. Additionally, in all hospitals, a specific QI initiative (the “tracer project”) was followed longitudinally for the duration of the fieldwork.

### Data collection

The data were collected over a 12-month period (April 2011 to April 2012) by researchers in the respective countries, according to common specifications [24]. Meso-level interviews were repeated in April 2012, in order to obtain data on the progress made during the year of the fieldwork. In all ten hospitals we interviewed professionals directly involved in quality improvement processes.

Data collection at the meso and micro level was undertaken using diverse techniques, namely interviews, documentation, non-participant observation of quality related meetings, and shadowing of professionals. The overall data collection process was guided by the six ‘QI challenges’ originally identified by the organizing for quality framework: structure, culture, emotions, politics, education, physical environment and technology [26]. Table 2 contains a brief summary of this framework.

**Table 2:** The organising for quality framework

Bate, Mendel and Robert [24] undertook a three-year international study that was explicitly designed to help practitioners and researchers understand the factors and processes that enable hospitals in the US and Europe (England and the Netherlands) to achieve-and sustain-high quality services for their patients. This original study took as its starting point that whilst technical factors, such as information systems, do play a major role in accounting for the quality ‘gap’, organisational and cultural factors are crucial in understanding how quality and safety improvement occurs. Based on in-depth, multi-level case studies of seven leading hospital, this research found that high-performing hospitals were able to achieve, and then sustain, high levels of quality because they recognised and had been extremely successful in addressing-on an ongoing basis-six common challenges. The six common challenges that were identified from the case studies were:	
1. structural - organising, planning and co-ordinating quality efforts	
2. political - addressing and dealing with the politics of change surrounding any QI effort	
3. cultural - giving ‘quality’ a shared, collective meaning, value and significance within the organisation	
4. educational - creating a learning process that supports improvement	
5. emotional - engaging and mobilizing people by linking QI efforts to inner sentiments and deeper commitments and beliefs	
6. physical and technological - the designing of physical systems and technological infrastructure that supports and sustains quality efforts	
The researchers represented these common challenges by means of a ‘codebook’ which took the form of a checklist that practitioners can use to identify where the organisational gaps in their local improvement efforts may lie and what they may need to do to address them.	

Source: QUASER research protocol [24]

We interviewed people in different roles such as members of the board responsible for quality, the director of structures dedicated to QI activity (e.g. director of risk or program infection control), micro-system managers and front-line professionals (doctors and nurses). We used a semi-structured interview guide, including topics appropriate for the role played by interviewees viewing them as key informants on how QI was being addressed at the various levels of analysis. Our interview guides can be accessed elsewhere [24]. Managers also provided any relevant documents deemed relevant to understanding the dynamics of the QI processes from an organizational perspective. A total of 383 interviews and 803 h of observations, including 207 h of meetings relating to QI, were conducted at the meso and micro level (see Table 3 for a summary of the fieldwork undertaken).

**Table 3:** Summary of fieldwork

Hospital	Meso-level			Tracer project			Micro-level		
	Ints.	Obs.	Mtgs.	Ints.	Obs.	Mtgs.	Ints.	Obs.	Mtgs.
The Netherlands a	37	90	25	9	65	19	9	130	26
The Netherlands b	36	100	31	15	31	7			
Sweden a	14	20	7	9	12	5	13	8	8
Sweden b	15	6	2	2	6	1			
England a	13	65	16	5	25	10	21	97	6
England b	24	20	7	5	10	3			
Portugal a	15	0	0	11	0	0	26	57	10
Portugal b	20	18	12	3	10	3			
Norway a	18	2	3	7	2	1	25	20	2
Norway b	25	2	1	6	7	2			
TOTAL	217	323	104	72	168	51	94	312	52

**Notes**: *Ints.* interviews, *Obs.* observations, *Mtgs.* meetings

As we began the fieldwork with collecting data at the meso-level (hospital), informants at this level indicated who would be the most valuable interviewees elsewhere in the organization; our criterion for selection was knowledge of how to improve quality in the hospital. Informants therefore included senior hospital managers, administrative staff, and health professionals with and without managerial responsibility. All interviews were audio-taped and transcribed in the native languages. Non-participant observation of key meetings and shadowing professionals are obstructive strategies of data gathering, so we used two strategies to minimize this potential effect: (1) we performed more than one shadowing activity with each professional, or we followed the same professionals in various meetings, in order to increase their confidence and adaptation to the presence of researchers, which also allowed the possibility of observing formal work routines as well as less routine processes; (2) we were careful in talking about our analytical dimensions in order to avoid inducing our expectations.

### Data analysis and coding

The data analysis procedure was conducted in three phases: 1-initial coding of raw data; 2- writing vignettes in order to achieve more process and cross-level oriented descriptions of QI in hospitals; 3- uncovering dualities using Gioias’s methodology [27].

In the first phase, all transcribed interviews, notes from observations of meetings and shadowing of professionals, and extracts from relevant documents were analyzed by the researchers in each country. This analysis, a conventional thematic analysis, was based on a code-book, previously agreed-upon among the researchers before the first stage of analysis. The initial code-book included the six organizational dimensions coming from the previously mentioned framework [26]. Using an interactive process, involving recurrently moving from data to interpretation, new emerging themes were assessed by researchers in QUASER consortium meetings. During this phase, it became clear that specific data from interviews, observation notes or documents did not grasp the complexity, richness and cross-level nature of QI occurring within study hospitals. This also led us to realize that most QI initiatives crossed several categories included in the initial code-book and that some initiatives seemed to represent contradictory instances, instead of data to include in more or less pre-defined categories, thus suggesting an additional interpretative approach.

In the second phase, each research team wrote a national report including vignettes, illustrating QI-related initiatives. It was our contention that, because of their inherent complexity, cross-level nature and vividness, vignettes best reveal the processes examined in the study. In each country, at least two researchers wrote the vignettes for each case study and, in some cases (e.g. Hospitals from The Netherlands), original informants read the vignettes in order to attest the accuracy of the descriptions. Table 4 contains examples of vignettes retained as illustrations of our analysis. Vignettes or short descriptions of cases written by researchers are being increasingly used as a source of evidence in organizational research [28–30]. Creating vignettes corresponds more to crystallization than to triangulation. Triangulation describes the credibility gains generated by the existence of various sources and types of data, different theoretical perspectives or different investigators converging on the same conclusion [31]. The notion of crystallization [32] assumes this multiplicity, without the concern of necessarily reaching the same conclusion, but rather can explore different interpretations and enlarged scope [33].

**Table 4:** Examples of vignettes representative of each second-order theme

**Plural consensus**	
*Tensions between different QI conceptualizations*	*Strategies to reconcile divergent views about QI*
What became apparent throughout the study period is a clear disconnect or tension between the publicly celebrated concept of quality, and the implicit, operational definition. There are several areas in which this contradiction appears apparent. Firstly, a tension appears to exist between finances and quality. Staff report how the quality issue quickly slipped off the agenda in the face of financial crises. This was despite the pressures exerted by the CQC to make immediate improvements. Although, the public narrative stresses that quality was at the forefront of the organisation, staff describe how tens of thousands of pounds are spent using a multitude of external consultants to assist the organisation in making financial savings. In contrast, only one external consultant was recruited to help the organisation improve upon its quality. At the same time staff describe how the organisation focused on improving upon the shortcomings flagged up by the CQC inspectors, but then suddenly lost this focus in the face of necessary financial savings **(England B**).	The informants often refer to the “quality puzzle” or more correctly “Safe Health Care – every time, all the time” which is another basic concept for quality improvement including patient safety. It is expressed as a puzzle with 14 pieces, where each piece contributes to the development of quality and patient safety and it illustrates the endeavour that good quality and safe care should permeate all treatment and care of patients. The project leader coordinating patient safety work follows up the clinical results in using the 14 pieces in the puzzle by using a matrix on the intranet. There she can follow to what extent each clinic works with the 14 issues which the clinics themselves are asked to judge. Clinical outcomes are measured, followed and visualized regularly on the intranet. (**Sweden A**).
**Distributed connectedness**	
*Pressures for independent work*	*Boundary spanners and multi-professional work*
While medical communities like the orthopaedic surgeons seem to learn from each other at daily meetings, a marketing and communication manager feels that ward managers are largely isolated from each other: “Well, I find it scandalous, how little we learn from each other. I’m trying to break down the walls, so that we can learn from each other. I want to be honest here, it’s crazy when you look at the wards. They don’t look any further than their own ward …because…sometimes, they might have thought up a good solution to a disturbing problem. If they shared that, everyone could do something with it (communications manager)” (**The Netherlands A**).	In the various meetings we have attended as observers with the quality team members and departmental directors and head nurses, it was mentioned several times that the quality team was always available to go to departments, that directors and head nurses could phone and ask a question or even appear in their office. Many times they arranged quality team visits to departments. At the same time it was emphasized that quality had to be part of every professional’s life “as naturally as putting on your gown in the morning when you start work.” (**Portugal B**).
**Orchestrated emergence**	
*General templates used to give sense to QI*	*Multiple and local organizational elements implementing QI*
The letter of assignment from the regional health authority (RHA) stipulates the following strategic areas related to quality improvement: 1) reduction of waiting lists, patient pathways, and deadlines, 2) user involvement 3) patient safety, and 4) quality measurements. The letter of assignment states that the health trust is responsible for taking part in the strategic regional quality effort; to participate in the steering group, the quality forum and the quality conference. Moreover the health trust should promote project proposals for strategic QI projects funded by the RHA; conduct patient experience surveys at the local level; report adverse events and use inspection reports for learning purposes; organize regular meetings across units to assess adverse events and use them for learning.. As such, the quality and patient safety targets set by the RHA generate learning arenas and learning activities within the hospital. (**Norway A**).	Productive Ward is construed as a bottom-up project, as it allows teams to choose locally relevant improvement objectives and tools. This is experienced as greatly motivating. Many participate because they feel finally able to regain control and ‘do’ quality improvement that goes beyond externally driven indicators. Nevertheless, the project is steered hierarchically: a steering group sets the overall agenda and decides on project continuation. The ward managers, who run the local working groups, are not part of the steering group. Contact between the steering group and ward managers is largely facilitated through the project leader, who communicates important outcomes of the steering group to the working groups. The executive director and the middle manager maintain contact with the ward-based work through site visits, when they hear about developments and bottlenecks. These visits are designed to demonstrate the relevance of the project work and also serve as qualitative mini-evaluations. These site visits link nurses and executive managers (not ward managers). The bottom-up project, while steered in a top-down fashion, tries to build on the motivational aspects of bottom-up work. (**The Netherlands A**).
**Formalized fluidity**	
*Indicators, targets and rules guide QI*	*Local judgment guides QI*
Some senior leaders and the vast majority of staff at middle management and frontline levels argue that their organisation had lost sight of the patients they care for. They saw the strong focus on efficiency measures, analysis of quantitative quality data and the commitment to meet national targets rather as a means to an end than the optimal approach to improve patient care. A senior leader reflected on this as follows: So demand from the general public and also demand from organisations. Endless streams of targets to try and achieve, which again, they are there for quality, so we have measuring incident rates of thrombosis, pressure sores, all these sorts of things, which is good, and nutrition analysis on the ward, but sometimes these things are … almost the analysis is the means to an end and I think we’re trying to do these things to ensure quality, not just to ensure that we’ve met the targets, and there seems to be a focus on that. And how do you get the balance when people have scorecards and they measure quality. I’m not sure the balance is right’. Most criticism of the hospital board came from clinical leaders at middle-management level. They were cynical about the obsession of senior leaders with assurance as they felt the hospital board denied recognising real problems. (**England A**).	Besides national quality norms, the hospital handles local quality norms aimed at positioning the hospital on the care market, as the local norms usually exceed the national ones. Some of these norms are being criticized as being too ambiguous, and at times, this leads to the official reduction of a local set norm, and elsewhere it results in local norms being ignored. Usually one tries to adhere to the hospital norm through a process that sometimes requires a little ‘tweaking’, as the case of bed occupancy highlights. The local norm states that all patients admitted with acute health problems remain on the acute entry ward for a maximum stay of 48 h and a ratio of 50% discharge to reduce the average duration of stay. The bed occupancy meeting is supposed to support compliance with this norm. The meeting takes place daily at 9.30 a.m. chaired by the admission manager, and all ward managers participate. All present ward managers focus their attention on a large wall screen that shows a matrix with numbers. All patients on the acute ward are discussed with regard to their diagnosis and preliminary therapeutic plans, and successively all those who have to undergo further hospital treatment are distributed to relevant wards. However two patients remain unplaced. Both are scheduled for surgery, but all surgical wards are fully booked. The paediatric ward has five vacant beds and the admission manager proposes placing them there until other beds become vacant. The manager of the surgery ward remarks that this would throw up serious questions about quality and safety, as these patients demand particular pre- and post-surgical treatment, such as adequate pain medication. Such care protocols, however, are not routinely used on paediatric wards. All ward managers present agree that this is an unsafe option. Thus, patient placement continues…(**The Netherlands A**).
**Patient coreness**	
*Centrality of patients in QI*	*Peripheral place of patients in QI*
There are a number of forums and activities regarding involvement of patients in the QI work, e.g. patient involvement in deciding on new treatment equipment, “learning cafés” where patients and related persons meet and discuss issues linked to their illness with other patients and relatives with the support of a resource person. There are also patient associations taking part in regular meetings regarding quality and patient safety at the hospital etc. The hospital performs patient surveys to get to know about the patients’ experiences. An example of concrete patient involvement is the rebuilding of the dialysis pavilion where patients participated in the choice of dialysis equipment and where the patients by now manage their own treatment assisted by care personnel only when needed (**Sweden A**).	The hospital had an internal policy which set out to employ 12 patient advisors to provide a critical view on service. Since two of them had left and had not been replaced, the hospital had only ten patient representatives at the time of our fieldwork. From an interview with a patient advisor, it was suggested that the senior management team had brought them under strict control, so that they had practically little impact, but were rather used as mediators between the hospital and the public. A patient advisor referred to this as: ‘Previously we could give an external view, an outsider’s view to the inside of here, now the change is that there is a temptation to ask us to reflect to the outside as the internal view, in other words we are more likely to be required to see things the hospital way than the patient way so I think this is actually a weakness of the system now’. (**England A**).
**Cautious generativeness**	
*Efforts to maintain systems´ integrity*	*Attempts to generate change from learning*
The informants talk positively about the team training activities in the maternity section, valuing interprofessional training activities as important QI learning arenas. Other important learning arenas are the morning meetings on the ward and different forums within the professional groups, both informal in form. During our observations we attended an informal lunch meeting among the paediatricians in which the experienced physicians contributed their experience and competence in the discussions about current challenges on the wards. The midwives can furthermore attend guidance offered by the Counselling Centre for mother and child in the maternity section. A midwife trained as a coach leads the group, and the attendees are coached about their performance and how to handle situations. Another learning arena is the weekly meeting about interpretation of STAN results (a type of monitoring of the infant during the birth process). The physicians bring cases or examples that have been difficult to monitor during delivery. In the meeting, physicians and midwives discuss the case, evaluate their performance and evaluate how they could improve performance. Our observations showed how the midwife room constitutes an important learning arena and an arena for experience transfer between professional disciplines and within the midwife group. In sum, the results show that the staff in the maternity section uses results from their practice for learning purposes and to improve their practical skills. (**Norway B**).	As part of a training session related with the subject of quality, the head nurse in the Intensive Medicine ward got together with three nurses and designed a monitoring project for intra-hospital and secondary transport of critical patients. The aim of the project was “to promote/ensure the safety of the patient/professionals in the transport of the critical patient, based on the premise that the level of care during transport should not be inferior to that in the original service, with the possibility of a higher level of care foreseen”. The project group created a formal document, presenting it to all the important internal stakeholders showing that the project contains all the information for the implementation including: evidence-based principles and practices to ensure the safety of this type of transport; an analysis of the most common type of incidents in this type of transport and a detailed description of the procedures used in the different phases; a description of the responsibilities of doctors/nurses/others; a detailed description of the equipment, medication and other support materials required for intra-hospital and secondary transport; three forms for recording each specific transport; procedural norms to be adopted by all nurses in the hospital cluster; guideline for the internal audit of the process. (**Portugal A**).

In the third phase, after vignettes were established as the major source of evidence, we used an analysis strategy guided by the Gioia methodology [27], an appropriate template to conduct qualitative analysis in organizational settings [34]. In line with the prescriptions of this approach, we started the data analysis by reading all the vignettes that were written by researchers and attached to each one a label attempting to capture the key process described. In other words, we discerned the first-order concepts. Unlike first-order concepts found in the literature [35], ours do not necessarily include terms originating directly from organizational actors, but from texts written by researchers. The expressions we used in our first-order concepts are intended to provide descriptive labels for conditions found in our data, a procedure previously applied when using this framework [36]. Then, we re-read all vignettes in an attempt to identify similarities between first order concepts. Using a constant comparison approach, we collapsed them into distinct clusters, or second-order themes, representing a more abstract level.

**Fig. 1:**
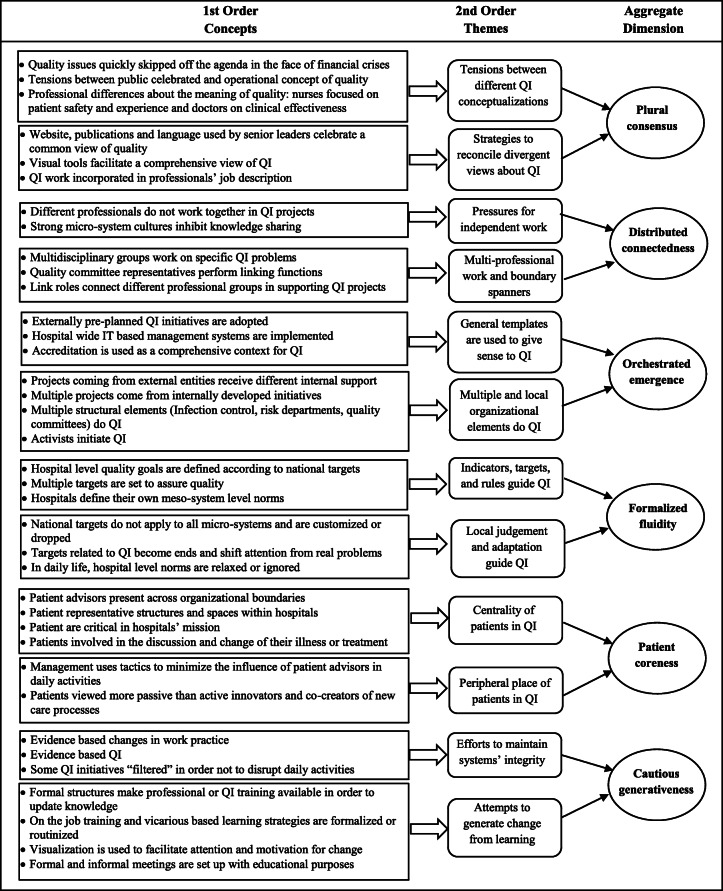
Data structure

**Fig. 2:**
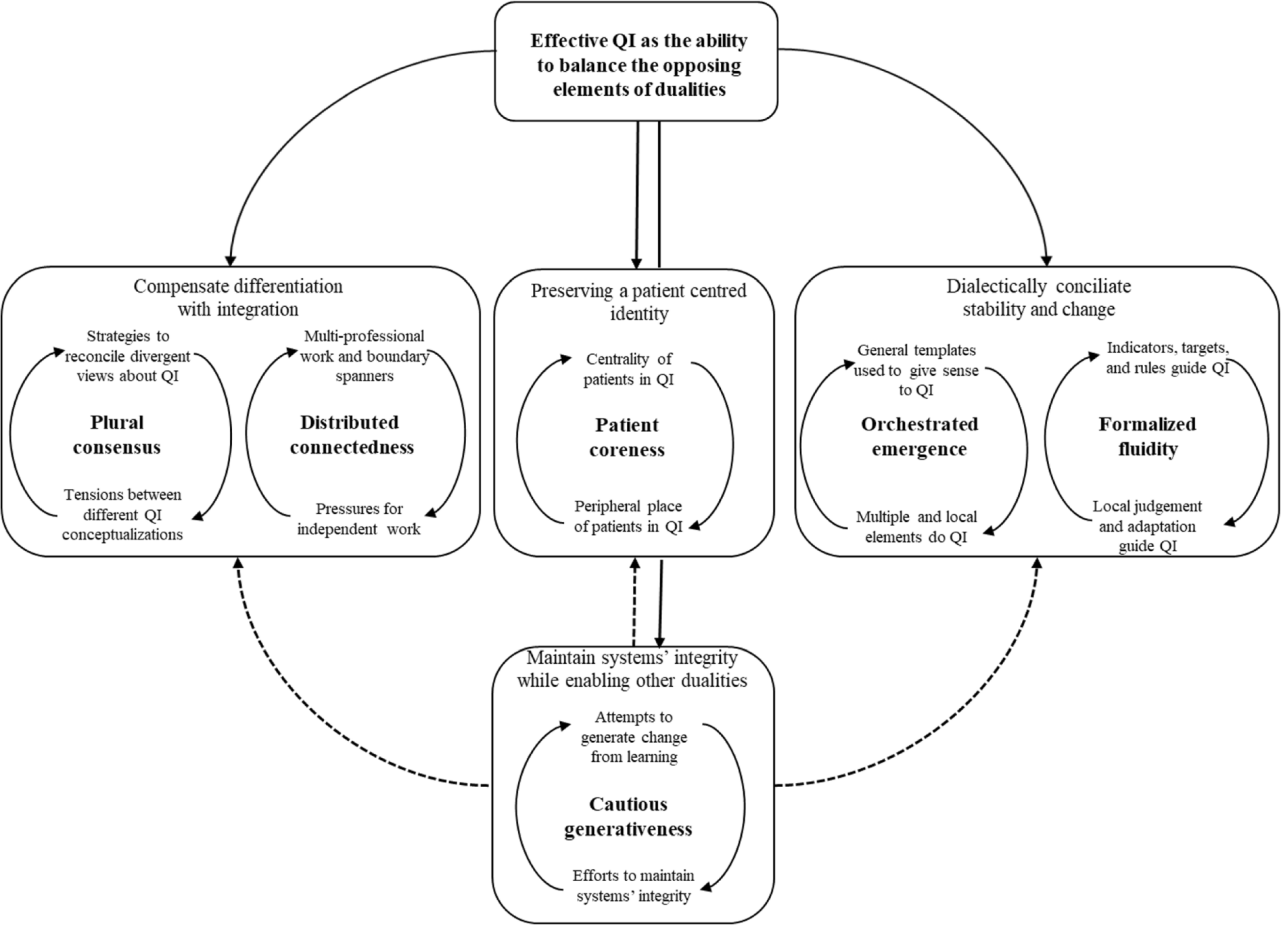
Organizational dualities involved in QI in hospitals

Finally, we distilled second-order themes into overarching theoretical dimensions, creating a data structure, a distinctive feature of this analytical approach because it provides a visual display of the progress from data to interpretation (Fig. 1). In this stage we used the literature to enrich our interpretative focus [37, 38]. Informed by the literature on dualities we attempted to juxtapose those processes representing oppositions, instead of searching for overarching dimensions representing labels of compatible organizational processes described in the second-order themes, thus highlighting the tensions of QI initiatives in hospitals. An example was the vignette: “In Norway Hospital A service users are represented in a user panel, the Quality Committee and in the steering committee of the QI programme. Patient representatives are also expected to be included at each step of QI projects. There are patient surveys and a mailbox to collect patient experiences on the wards”. This content can be classified as a political, educational, or emotional challenge, according to the initial code-book [39]. (Anderson et al., 2019). But it can also be interpreted as a situation illustrating the centrality of patients in QI, a position that can be opposed to a peripheral place occupied by patients, one of the dualities we identified. Finally, after consulting additional literature about the possible articulation of the emergent concepts, we elaborated on the nature of the relationships between dualities proposing that effective QI can be seen as the ability to balance the opposing elements of dualities (Fig. 2).

Still in line with the practice of the Gioia methodology [40], in addition to the sequential steps, the trustworthiness of our analysis was reinforced by having multiple researchers assessing the process of assignment of codes and subsequent categorization. During the entire analytical process, we moved to the next stage when broad agreement between researchers was reached.

## Results

The data structure produced by our analytical approach described above is represented in Fig. 1. Aggregate dimensions and second order-themes are used to organize the presentation of our findings. Overall, our analysis revealed six dualities involved in the search for improved care in study hospitals, each one representing the integration of oppositional demands: plural consensus, distributed connectedness, orchestrated emergence, formalized fluidity, patient coreness and cautious generativeness. For illustrative purposes, we include a vignette integrated into each second-order theme (Table 4).

### Plural consensus

Our data reveal the tension between the second-order themes describing the existence of different conceptualizations of QI in hospitals and the strategies for reconciling these divergent views. In our view, this tension between different conceptualizations of QI and compensatory strategies to settle them are the two sides of the same coin, the duality of plural consensus. Plural consensus is the ability to recognize and appreciate differences and the richness that comes from diversity, combined with the cross-understanding among different perspectives that is required to support the coordination of QI activities.

#### Tensions between different QI conceptualizations

Our data reveal that in European hospitals QI is pursued in a contested terrain of the meaning of quality and the place of improvement with regard to other priorities. The diversity of meanings is largely shaped by competing goals, namely the search for efficiency in the context of generalized cost containment evident in European hospitals. But these tensions are also evident between the publicly celebrated views of quality - often in line with institutional understandings - and operational conceptualizations, often more nuanced and practice-oriented. Finally, different professional groups espouse meanings of quality anchored in their occupational identity; nurses tend to emphasize patient safety and patient experience, while doctors are mainly focused on clinical effectiveness, and managers tend to approach quality on the basis of performance indicators that can be routinely reported and monitored.

#### Strategies for reconciling divergent views about quality

Despite the evident variability of what hospitals do and think regarding quality, and the potentially negative impact this might have on the shared understanding required to accomplish coordinated work, we found a number of strategies in place in hospitals to deal with the diversity of meanings attached to quality. We found comprehensive views of quality expressed on hospitals’ websites and internal documents, as well as explicit communication strategies used by top management leaders that were intended to foster a shared understanding of quality. Some hospitals developed visual tools, for instance using a puzzle metaphor to facilitate an integrated view of quality that was available on the hospital intranet to accommodate multiple, local QI initiatives and monitoring. Data also reveal how hospitals tried to integrate QI into professionals’ daily practice, for example, by explicitly incorporating quality work into job descriptions, thus fostering a common view of professionals’ role as improvement agents.

### Distributed connectedness

In line with the notion of plural consensus, our data also show the existence of pressures for both independent and multi-professional/boundary spanning work. These two second-order themes were complementary processes in the hospitals studied, and both are required to achieve effective QI. We consider perceiving them as two distinct but complementary processes to be a duality we label ‘distributed connectedness’. Together, plural consensus and distributed connectedness challenge hospital management to deal with tensions between requirements for both differentiation and integration.

#### Pressures for independent work

Our data show not only different conceptualisations of quality, but also pressures that lead to uncooperative work in providing care. Despite the generalized acknowledgement that the delivery of care - as well as its improvement - is a highly interdependent activity, our data reveal that hospitals struggle to face this challenge. In fact, not only do different professionals tend not to work together in QI initiatives but strong micro-system cultures and specificities inherent to work hinder the transfer of good practices and QI knowledge from one unit to another.

#### Multi-professional work and boundary spanners

Despite the evidence of strong pressures for independent work, influenced by professional identities and strong micro-system cultures, our data also reveal several routines and organizing solutions intended to counterbalance that tendency. Sometimes, multi-professional project groups are established in order to analyze specific QI problems or to help in the local customization of supra-hospital initiatives. We also found evidence of boundary spanning roles occupied by representatives of formal structures (aimed at connecting different professions or micro-systems), or more temporary linking roles fulfilled by internally recognized individuals who were able to perform similar connecting ‘work’. The function of these activities is to create a relational infra-structure in which QI initiatives can be situated and enacted.

### Orchestrated emergence

In the ten study hospitals, we found that QI is being enacted by general templates aimed at giving a collective sense to QI efforts but also by multiple and local actors simultaneously adhering, opposing or customizing these general initiatives, or simply coming up with local changes aimed at providing better care. We name the ability to see these two processes - not independent from one another but complementary - as orchestrated emergence capability.

#### General templates used to give sense to QI

Our data show that hospitals often become involved in QI in response to initiatives devised externally by agencies, such as the World Health Organisation, the Ministry of Health or county-level official bodies. Usually, these programs have a prescribed approach to implementing the QI program, even if they allow for some local adaptations and tailoring. Other general templates to conduct QI can come from national or regional accreditation programs, indicators for benchmarking and clinical guidelines. Comprehensive IT systems are also used to translate supra-hospital QI templates and targets down to the hospital level. These approaches are used as sense-giving devices fostering a comprehensive framework within which to conduct QI.

#### Multiple and local organizational elements implementing QI

In contrast to the use of general templates to guide the implementation of QI, the hospitals studied also became involved in a number of internally shaped or ‘home grown’ initiatives. In fact, even when projects are externally designed by influential institutions and the top management formally adopts them, often by buying into a project, not all micro-systems, professional groups or other powerful actors adhere in the same way. The degree of involvement may be dependent either on the degree to which the change implied is likely to lead to improvements that do not completely depart from the status quo or whether they enhance individual, professional or micro-systems’ identities. Moreover, changes introduced in order to improve a particular aspect of quality are often initiated by individuals trying to adopt best professional practices or activists who intrinsically want to improve care. Finally, formal organized structures designed to improve quality or specific aspects of quality (for example, quality committees, infection control teams and risk departments) also start attempts to improve care, initiating change processes that are often not fully implemented, potentially leading to the demotivation of professionals.

### Formalized fluidity

Our data reveal that in order to pursue QI, hospitals rely on management systems based on indicators, targets and rules to guide their efforts, and at the same time rely on local judgment and adaptation. We view these two tendencies as complementary and constitutive of the capability we name formalized fluidity. In conjunction with orchestrated emergence previously described, formalized fluidity refers to a challenge faced by hospitals striving to improve care, namely the ability to manage the tension between control and flexibility.

#### Indicators, targets and rules guide QI

Due to overwhelming concerns with efficiency, the nature of the financing model of hospitals or accountability requirements, study hospitals established quality indicators, targets and rules to help them attain certain levels of performance, thus responding to contextual demands. Very often, alongside such externally influenced indicators and targets, hospitals also defined their own metrics in order to monitor quality. Some hospitals also defined rules aimed at guiding decision-making towards goal attainment. The result was the creation of a management system - often objectified as a “tableau de bord” or a “scorecard” formed of multiple indicators that can be monitored - and targets whose attainment was intended to lead to rewards and rules or procedures that guide daily action toward goals.

#### Local judgment and adaptation guides QI

Despite the influence of the indicator-target-monitoring management system in determining the dynamics of hospital QI efforts, especially at the meso-level, a shift of attention to the micro-system level led us to uncover a rather different reality. Our data show how nationally developed quality targets do not apply to all micro-systems within the hospital, and how these units customize, drop or choose distinct benchmarks for indicators. In addition, informants told us how an excessive focus on QI targets becomes an end in itself and leads to avoiding the challenge of solving real quality problems. Finally, our data highlighted how hospital-level rules can be relaxed or even ignored by professionals in order to accommodate the dynamics used by micro-systems to solve their own specific problems.

### Patient coreness

Our data reveal the existence of recurrent practices giving patients a central role in QI, and other routines that locate patients at the periphery of QI efforts. Central and peripheral places given to patients in pursuing QI are the two second-order themes that together make up the duality of patient coreness. We view these two types of routines and organizational arrangements as two poles of the same dimension.

#### Centrality of patients in QI

In line with a current trend towards patient centeredness, study hospitals enacted several routines and organizational solutions that gave patients a central role in QI. In fact, some hospital mission statements contained patient involvement as a critical element. In some hospitals patient advisors existed across organizational boundaries and are consulted to assess current work, in others patients’ representative structures could be located inside hospitals’ facilities, and sometimes patients were involved in multidisciplinary groups in order to discuss their conditions or even input to changes in the way treatments are provided.

#### Peripheral place of patients in QI

In contrast with such arrangements and routines intended to give patients a central role in improving care, hospitals also engaged in practices to relegate patients to a more superficial position. Sometimes in a subtle way, professionals continued to view patients more as objects of their intervention than active contributors to innovation and co-creators of improved care processes. Additionally, we found management practices that - although accepting the role of patient advisors - were performed in order to prevent patients having any real influence in decision-making.

### Cautious generativeness

We found evidence that hospitals simultaneously undertook efforts to maintain systems’ integrity whilst, on the other hand, attempting to induce change from learning activities. This duality reflects the tension between exploiting the already existing and proven knowledge versus exploring new ideas. Because it implies the ability of hospitals to deal with both a system’s protection of integrity as well as change, we call this duality cautious generativeness.

#### Efforts to maintain systems’ integrity

We gathered plenty of evidence of professionals excited about how their practices were incorporating new knowledge, often emerging from carefully designed research processes and translation activities. Most of the externally adopted QI programs we encountered were evidence-based and usually pilot-tested before being made available as improvement templates. Our observations also revealed micro-system leaders and policy advisers acting as filters, responding directly to contextual pressures or locally emergent initiatives but also actively deciding what changes were important enough to be actually implemented and what could be considered inopportune, irrelevant or too overtly disruptive.

#### Attempts to generate change from learning

In addition to efforts to carefully protect systems’ integrity, study hospitals also engaged in more active experimentation and learning. We found formal structures in place in most hospitals to guarantee that professional knowledge was updated, even if some informants expressed limitations due to financial constraints. We also observed very routinized and conventional on the job training and vicarious learning-based strategies (not unexpected given the tacit nature of some clinical knowledge). Our observations also revealed the existence or more or less routine-based formal and informal meetings explicitly held for educational purposes.

## Discussion

The purpose of this research was to identify the dualities faced by hospitals in their efforts to improve the quality of care. Our findings revealed the existence of six dualities involved in the search for improved care in hospitals describing ways of dealing with oppositional demands: plural consensus, distributed connectedness, orchestrated emergence, formalized fluidity, patient coreness and cautious generativeness. Each of these dualities points to the need to deal with a tension, even if the nature of this tension varies between dualities and sometimes becomes a major challenge to be faced by hospitals in their quest for improved care. Figure 2 depicts our proposed description of the meta-capabilities to which hospital managers should attend to improve quality.

Plural consensus describes hospitals’ need to deliver quality through the management of tensions shaped by different quality understandings through the implementation of strategies to conciliate them. According to our data, for instance, in conceptualizing quality, nurses, doctors and mangers tend to emphasize different dimensions of quality, while some hospitals developed visual tools to facilitate an integrated perspective of QI. The central idea is not the need to develop a homogeneous perspective of ‘quality’ in a context of high diversity [41] but rather to recognize that generalized common understanding is an enabling condition for coordinated action [42]. Plural consensus is achieved by maximizing members’ knowledge about QI and promoting cross-understanding amongst members of different groups [43], i.e., maximizing the extent to which people belonging to different groups have a precise understanding of the mental models of members of their own and other groups, and are able to notice differences and communalities, eventually linked by a common supra-level purpose.

Distributed connectedness labels hospitals’ need to improve service quality by recognizing pressures for independent work which characterizes them, and the use of compensatory solutions of multi-professional work and boundary spanners. Our data reveal the existence of strong micro-system cultures hindering the QI knowledge exchange within organizations, but also multi-professional groups and boundary spanners nurturing a strong relational network supporting QI. Distributed connectedness stresses the role of patterns of relationships as a foundation of QI, as a platform of pre-existing relationships affects the impact of QI leadership [44] as well as daily activities. Additionally, on a daily basis, we can argue that a rich multilevel and multi-professional relationship pattern is an organizational competence conducive to better patient experience, patient safety and clinical effectiveness, because it allows all professionals that constantly make decisions about patients to collectively engage in frequent, rigorous, timely and problem-solving oriented information-sharing [45].

Plural consensus and distributed connectedness capabilities are of a compensatory nature (Fig. 2). Hospitals create organizing solutions and routines to reach some degree of consensus amongst plural views of quality and to achieve connectedness in a highly distributed context. Plural consensus and distributed connectedness are capabilities developed to achieve an optimal level of balance between differentiation and integration [46], a central challenge faced by health care organizations wanting to improve [47].

Orchestrated emergence refers to hospitals’ capability to improve quality by being able to combine general templates that give sense to QI initiatives with multiple enterprises originating in specific actors who want to deliver better service quality. Our data reveal that QI initiatives can come from general templates coming from external entities, what contrasts with locally developed initiatives. Orchestrated emergence is the capacity to centrally coordinate and guide multiple QI initiatives toward institutionalization, i.e., to facilitate the process by which what is learned is embedded in routines, culture, and information systems and physical space until it is challenged again [48, 49]. Orchestrated emergence contributes to QI effectiveness by capturing the change potential coming from a wide range of motivated actors interested in doing better work, and gives consistency to this process by integrating it into a more comprehensive strategy for QI as framed through general templates.

Formalized fluidity describes hospitals’ capability to achieve improved quality by relying on management systems based on indicators, targets and rules to guide QI and by promoting both local adaptation and professional judgment. As shown by our data, hospitals tend to rely on formal indicators and rules to guide QI but, at the same time, some micro-systems customize, relax or ignore formal indicators or rules to better reflect local dynamics. Formalized fluidity contributes to QI effectiveness by helping hospitals to deal with the classic problem of reconciling control and flexibility [50] by combining the benefits of consistency coming from standardized procedures, guidelines and indicators - fundamental in interdependent work as they increase behavioral predictability among all agents - with the benefits of adaptability to specific contexts coming from autonomous individuals using their judgment to find new answers to changing conditions of their task [51]. This capability will help to avoid the “gaming of data and goals”, a sign of a vicious cycle of over-riding goals, misallocation of resources, distracted attention, and the consequent failures and threats [52].

Unlike plural consensus and distributed connectedness capabilities which share a compensatory nature and address the differentiation-integration concern, orchestrated emergence and formalized fluidity are of a dialectical nature, in the sense that clear contradictory processes are combined in a supra-level capability of dealing with both stability and change (Fig. 2). This is in line with evidence of some organizations being highly effective precisely because they are able to simultaneously emphasize opposite cultural orientations of innovation and change and - at the same time - stability and control [53].

Patient coreness is hospitals’ capability to devise proper organizational elements or routines that locate patients as central players in pursuing improved quality of care. Our data show a clear tension between initiatives giving voice to patients, but also others, perhaps subtler, devised to assure that patients do not have a strong influence on decision-making. Although common definitions of quality are patient-centered, and a growing amount of literature suggests that patients can perform relevant functions in improvement, the assessment of public and patient roles in improvement reveals a low effect [54]. To move patients to the core of hospitals represents the process linking QI practices involving patients to other organizational elements. In this sense, coreness represent more or less rich webs of connection among organizational elements [55] such that more central organizational elements gain inertia when they are connected with many other [56] rendering change a more difficult task. Consequently, core elements are not easily skipped in face of constraints or put aside by contextual tactics. If we accept that the purpose of hospitals is to contribute to patient well-being, then patient coreness also performs a function of organizational identity protection.

Cautious generativeness is the hospitals’ capability to protect the integrity of current activities by introducing very incremental and tested changes and at the same time providing ample opportunities to think about change from learning. But this generalized learning is conducted in a careful way because trial and error-learning is impossible in daily activities [57] and too much of a learning orientation can undermine performance in complex environments [58]. Cautious generativeness enables QI by allowing hospitals to continuously incorporate new knowledge into daily activities, facilitate information sharing, without disrupting current work or organizational structures. Cautious generativeness echoes propositions about organizations needing certainty in uncertain environments [59]. Consequent attempts to ‘seal off’ core technologies from external influences equates to views of a learning system as one that allow change without strongly threatening systems’ identity [60]. Cautious generativeness is a very different capability from the others we have identified because learning from experience is the mechanism through which other capabilities are developed and thus allow hospitals to both perform daily patterns of activities and search to implement new processes [61]. Like patient coreness, cautious generativeness also contributes to protecting the essential purpose of hospitals and thus performs an identity maintenance function.

### Implications for quality improvement approaches

Compared with CFIR [10] or MUSIQ [11] meta-models, the set of dualities we identified portray a very different picture about how QI should look in hospitals. If we follow the proposition of the paradox approach, according to which tensions become more salient in plural organizations that are required to change permanently and face resource shortage, hospitals fulfill these characteristics completely. By searching for tensions and corresponding reconciling meta-capabilities, instead of providing a comprehensive framework to understand what organizational elements are potentially relevant in improvement, or guiding the implementation of more effective QI projects, the emphasis on system level capabilities, tensional in nature, shifts the attention from specific QI projects to see QI as an overriding management challenge, entailing the need for compensatory strategies for the integration and differentiation of quality work and balancing strategies for change whilst maintaining systems’ integrity.

Appropriate management strategies entail the acceptance of paradoxes as vital ingredients of high performance [62] and an invitation to creative problem solving [63]. After being accepted, paradoxes can be managed by a strategy involving differentiation activities, or creating formal and informal activities targeted at each element of the paradox (e. g. separate structural elements, distinct leadership roles, different learning times) combined with integration activities accommodating both elements (e. g. boundary spanners, all-embracing strategic aspiration, assigning integrative roles to leaders, complex cultures). Specific work targeted at both differentiation and integration will support appropriate paradox management enabling organizational sustainability and managers are the key actors responsible for these organizing activities. Instead of thinking about QI as initiatives to generate fast changes in the provision of care [64] we invite managers to carefully craft a strategy to develop a set of meta-capabilities able to deal with the dualities we identified, thus contributing to sustained healthcare quality.

Additionally our study calls for a move from the usual sequential thinking about quality (e.g. Plan-Do-Study-Act methods) to a more Janus-like approach indicating an ability to notice the simultaneous operation of two opposing ideas or concepts [65]. Existing approaches to QI are usually based on assumptions of alignment and coherence about organizational dynamics and our study significantly challenges these assumptions by highlighting the inherently tensional nature of health care organizations. In our view, sustained QI is best achieved when leaders hold inconsistency, contradictions and tensions. This requires a shift in the management mind-set, to one that notices and embraces rather than ignoring or refusing opposing demands.

In this context, a key challenge is to craft a management development program aimed at promoting the emergence of this Janusian thought style. Combined with the results of this study, paradox literature provides useful initial elements for the design of such a development program [66]. Among the aspects requiring change, we can mention: valuing dualities as vital elements of organizations, especially those operating in plural, uncertain and complex contexts; becoming actively involved in identifying tensions between two opposite demands; creating separate agendas to develop different sides of the duality; learning how to cope with the rising anxiety and defensiveness that ensue from leaving a more linear way of thinking; acting consistently inconsistently; holding multiple strategies and identities concurrently.

### Limitations and future research

Our study has important limitations. Although the research involved ten case studies from five countries, other hospitals embedded in other national contexts may have developed specific patterns of organizing arrangements and routines to enact QI. On the other hand, while intentionally designed to repeat meso-level interviews 1 year after the initial fieldwork began in each hospital in order to trace changes, the time frame was not long enough to grasp important changes affecting dualities.

Besides addressing these limitations, future research could examine to what extent other health care organizations, in addition to hospitals, facing similar change, resource, and institutional circumstances generate the dualities we identified. More importantly, future research could develop and test the validity of a management development program combining educational and field activities targeted at enabling managers to embrace contradictory tensions and, thus, make sense of QI challenges in new ways.

The exploratory nature of our study, coupled with the challenges involving the classification of hospitals as more or less performing in QI, did not allow us to establish a relationship between the ability shown by hospitals in dealing with the dualities and their performance in terms of QI. Future research should develop a measure of this ability and assess the strength of the relationship between these dimensions. Conducted at the level of the micro-systems, this research could investigate the nature of the relationship of each duality with QI performance or, perhaps more importantly, the existence of different configurations of dualities explaining QI performance [67, 68], an enquiry that could draw on a qualitative comparative approach [69].

## Conclusions

This study has sought to apply a dualities perspective in studying the organizing arrangements and routines used by hospitals in relation to QI. The findings provide evidence of the existence of the dualities of plural consensus, distributed connectedness, orchestrated emergence, formalized fluidity, patient coreness and cautious generativeness. Overall, the development of these meta-capabilities helps hospital managers to deal with the fundamental challenges inherent to hospitals’ dynamics so as to reach optimal levels of balance between differentiation and integration, stability and change, and learning and preserving identity. This study invites hospital managers to adopt a Janusian style of thought required for the development of system-level capabilities which are fundamental to handle the opposing demands involved in QI.

## Data Availability

All data is included in the five country reports and available from the first author.

## Abbreviations

QI: Quality improvement
CFIR: The Consolidated Framework for Implementation Research
MUSIQ: The Model for Understanding Success in Quality
QUASER: Quality and Safety in European Hospitals
IT: Information Technologies
